# Pharmacological Interventions Targeting Pain in Fibrous Dysplasia/McCune–Albright Syndrome

**DOI:** 10.3390/ijms24032550

**Published:** 2023-01-29

**Authors:** Anthony Tucker-Bartley, Daryl J. Selen, Emma Golden, Raquel van Gool, David Ebb, Michael Mannstadt, Jaymin Upadhyay

**Affiliations:** 1 Department of Anesthesiology, Critical Care and Pain Medicine, Boston Children’s Hospital, Harvard Medical School, Boston, MA 02115, USA; 2 Department of Anesthesiology, Critical Care and Pain Medicine, Massachusetts General Hospital, Harvard Medical School, Boston, MA 02114, USA; 3 Division of Endocrinology, Department of Medicine, Warren Alpert Medical School of Brown University, Providence, RI 02903, USA; 4 Department of Pediatric Hematology Oncology, Massachusetts General Hospital, Harvard Medical School, Boston, MA 02115, USA; 5 Endocrine Unit, Massachusetts General Hospital, Harvard Medical School, Boston, MA 02115, USA; 6 Department of Psychiatry, McLean Hospital, Harvard Medical School, Belmont, MA 02478, USA

**Keywords:** fibrous dysplasia, McCune–Albright syndrome, pain, analgesia, bisphosphonates, denosumab

## Abstract

Fibrous dysplasia (FD) is a rare, non-inherited bone disease occurring following a somatic gain-of-function R201 missense mutation of the *guanine-nucleotide binding protein alpha subunit stimulating activity polypeptide 1* (*GNAS)* gene. The spectrum of the disease ranges from a single FD lesion to a combination with extraskeletal features; an amalgamation with café-au-lait skin hyperpigmentation, precocious puberty, and other endocrinopathies defines McCune–Albright Syndrome (MAS). Pain in FD/MAS represents one of the most prominent aspects of the disease and one of the most challenging to treat—an outcome driven by (i) the heterogeneous nature of FD/MAS, (ii) the variable presentation of pain phenotypes (i.e., craniofacial vs. musculoskeletal pain), (iii) a lack of studies probing pain mechanisms, and (iv) a lack of rigorously validated analgesic strategies in FD/MAS. At present, a range of pharmacotherapies are prescribed to patients with FD/MAS to mitigate skeletal disease activity, as well as pain. We analyze evidence guiding the current use of bisphosphonates, denosumab, and other therapies in FD/MAS, and also discuss the potential underlying pharmacological mechanisms by which pain relief may be achieved. Furthermore, we highlight the range of presentation of pain in individual cases of FD/MAS to further describe the difficulties associated with employing effective pain treatment in FD/MAS. Potential next steps toward identifying and validating effective pain treatments in FD/MAS are discussed, such as employing randomized control trials and probing new pain pathways in this rare bone disease.

## 1. Introduction

Fibrous dysplasia (FD) is a rare (prevalence between 1/100,000 and 1/1,000,000) genetic, non-hereditary disorder characterized by the formation of craniofacial or axial–appendicular skeletal lesions [1,2,3,4,5]. FD arises due to a somatic activating mutation in the *GNAS* gene, which encodes the alpha subunit of the Gs protein [3,4]. This results in the constitutive stimulation of adenylyl cyclase and production of cyclic AMP, leading to the proliferation of skeletal progenitor cells and FD lesions, which are characterized by fibrous tissue interwoven with normal bone, the accumulation of non-mineralized bone, and an increase in osteoclastic activity [6,7]. FD can occur in single (monostotic FD) or multiple (polyostotic FD) skeletal locations. Skeletal lesions in combination with café-au-lait skin hyperpigmentation, precocious puberty, and other endocrinopathies define McCune–Albright Syndrome (MAS) [2,8].

Patients with FD/MAS that develop craniofacial skeletal dysplasia may experience a range of neurological and ophthalmological manifestations such as vision loss, hearing loss, headache, migraine, and orofacial pain [9,10,11,12,13]. In comparison, FD of the axial–appendicular skeleton can lead to bone pain, vertebral deformities, and fractures [14,15,16]. Neuropathic-like pain arising in a subgroup of patients with FD/MAS points to the need for a more individualized implementation of analgesic strategies that incorporate, for example, gabapentinoids and other treatments that may relieve neuropathic pain (e.g., antidepressants or other anticonvulsants) [17]. Effective pain treatment remains a major clinical challenge in FD/MAS, an outcome driven by a lack of understanding of the pain mechanism active in the disease, the variable expression of pain phenotypes across patients, and the heterogenous nature of FD/MAS [14,18]. Adding to the complexity of pain treatment in FD/MAS is a well-known dissociation between skeletal disease burden and the intensity of pain reported by patients [10,16]. Thus, surgical or pharmacological approaches that directly target the morphological properties or structural matrix of FD lesions may not consistently translate to an amelioration of pain.

Here, we discuss pharmacological therapies, mainly bisphosphonates and denosumab, prescribed off-label to treat osteolytic bone lesions and pain. We examine the known mechanisms of action that are hypothesized to yield pain relief and the current level of evidence demonstrating their respective analgesic efficacy. We describe two patients with FD/MAS to highlight the challenges in pain management. The current lack of effective pain management for this disease calls for a more rigorous evaluation of existing therapies; for example, in randomized clinical trials (RCTs), and also, for the identification of novel and targetable pain mechanisms active in FD/MAS. Our working hypothesis is that pain in FD/MAS is a complex, dynamic, and heterogeneous process, whereby the neurobiological aspects of pain can evolve over time, requiring an adaptable approach to effectively treat it.

## 2. Therapeutics for Treating Pain in FD/MAS

Bisphosphonates: Bisphosphonates are prescribed to patients with FD/MAS with the goal of suppressing hyperfunctioning osteoclasts, which drive the development of osteolytic bone lesions [4,6,7]. Bisphosphonates are pyrophosphate analogues that bind strongly to bone mineral hydroxyapatite [19], are taken up by osteoclasts [20], and inhibit their activity, thereby reducing bone resorption [21,22,23] and increasing bone mineral density [24]. Although bisphosphonates are not approved by the Food and Drug Administration (FDA) for FD/MAS, they are routinely used in clinical practice [8,25]. The current body of evidence guiding bisphosphonate use in FD is comprised largely of case reports, case series, and a single RCT [26] (Table 1). These studies have varied endpoints ranging from bone turnover markers (BTMs, e.g., collagen telopeptides, osteocalcin, hydroxyproline, pyridinoline, alkaline phosphatase (ALP)), lesion properties (i.e., lesion size or activity) as measured by computed tomography (CT), magnetic resonance imaging (MRI), or positron emission tomography (PET), fracture rate, bone mineral density measured by dual energy X-ray absorptiometry DXA, bone pain, and functional status and mobility.

In a case series of twenty patients with FD, intravenous pamidronate 180 mg given every six months for three years showed the filling of osteolytic lesions and cortical thickening on radiographic imaging in nine out of twenty patients [27]. There was a 71% reduction in urine levels of type 1 collagen C telopeptide (CTX) following treatment in ten of twenty patients. Hypocalcemia was common despite all patients receiving supplemental calcium and vitamin D throughout the study period [27]. Thirteen out of twenty patients complained of bone pain prior to treatment initiation with an average pain rating of 2.8 on a scale of 0 to 4. At six months following treatment, 8 of 13 had complete pain relief, while 5 had partial pain relief that was sustained through 36 months. Eight patients had relapse after 36 months. Patients also reported reduced numbers and severity of painful bone sites [27].

Newer, nitrogen-containing bisphosphonates have since become available, in oral and intravenous formulations. In the single double-blind RCT available to date, oral alendronate was evaluated in FD patients with at least two skeletal lesions and age greater than 12 years old [26]. Patients who had taken bisphosphonates within one year prior to trial enrollment were excluded from the study. Alendronate or placebo was given to 40 patients over a 24-month period (6 months on and 6 months off). Dosing was weight-based; 40 mg for patients > 50 kg, 20 mg for patients 30–50 kg, and 10 mg for subjects 20–30 kg. The endpoints were alendronate’s impact on BTMs (e.g., type 1 collagen cross-linked N-telopeptide (NTX) and osteocalcin), bone pain, and areal bone mineral density of the FD lesions. There was no significant difference between alendronate and placebo with respect to BTMs, functional walk speed or distance, number of fractures, or pain.

In a recent single-centered retrospective study, 8 out of 13 (62%) patients with FD and associated bone pain were treated with zoledronate, all of whom reported partial or complete relief of bone pain [28]. This improvement was associated with decreased analgesic usage, and in some cases, improvement in physical function and mobility. The other five patients declined zoledronate due to potential side effects or were deemed inappropriate candidates based on clinical judgement. Five out of thirteen patients treated with zoledronate had decreased BTMs. Muscle aches and fever occurred in six treated patients, but none developed hypocalcemia or renal toxicity [28].

Interestingly, case studies report that some patients have had successful responses to one bisphosphonate but not to another. For example, a 32-year-old patient with cranial FD lesions in the middle and posterior skull with associated occipital headaches failed to respond to IV pamidronate but noted resolution of headaches, decreased ALP levels, and improvement in craniofacial lesions on computed tomography (CT) one month after treatment with zoledronate [29].

In a retrospective study, 22 patients with FD evaluated over the course of one year had reductions in BTMs [30]. More specifically, ALP levels decreased 34% for those treated with zoledronate, 30% for those treated with alendronate, and 23% for those treated with pamidronate. Limitations of prolonged bisphosphonate use include the potential adverse effects osteonecrosis of the jaw and atypical femoral fractures, which have been reported in patients with osteoporosis, and its accumulation in bones, which might limit their use in women of childbearing potential [31,32,33,34].

Denosumab: Denosumab is a humanized monoclonal antibody designed to target the receptor activator of the nuclear factor kappa-B (RANK) ligand, which plays a key role in the activation of osteoclasts, the main bone-resorbing cells. Normal osteoclast differentiation relies on the binding of the RANK ligand (RANK-L, secreted by osteocytes and osteoblasts) to its receptor RANK (located on the cell surface of osteoclasts and osteoclast precursors), which triggers the differentiation of osteoclast precursors and the activation of mature osteoclasts [35]. Denosumab blocks the ligand–receptor interaction by binding to and inactivating RANK-L [35]. At present, denosumab is approved by the FDA for use in post-menopausal women with severe osteoporosis (60 mg subcutaneous every six months as Prolia™), prevention of skeletal-related events in patients with bone metastases, and giant cell tumor (120 mg every 4 weeks with additional 120 mg dose on days 8 and 15 of the first month as XGEVA™ [36]). In patients with osteoporosis and a recent fracture, denosumab was shown to achieve pain control two weeks sooner and to increase bone mineral density further beyond baseline compared to alendronate given to similar patients [37]. There was no significant difference in serum BTMs (i.e., NTX) or rate of adverse events, such as hypocalcemia [37].

RANK-L inhibition is also an attractive treatment modality for FD particularly because FD lesions show high RANK-L expression [38]. In animal studies of FD/MAS, denosumab treatment arrested the expansion of established lytic bone lesions and prevented the development of new foci [39]. Off-label treatment with denosumab has been described in case reports with strong analgesic effects noted shortly after the induction of treatment [40,41,42,43,44,45] (Table 1); however, no RCT has been reported. Most of these reports used doses of denosumab at 60 mg subcutaneously every three to six months or 120 mg every six months, often following a loading dose during the first four weeks of therapy.

Several case studies report improvements in FD pain after the administration of denosumab [42,45,46], though the reemergence of bone pain despite continued suppression of BTMs has been observed between denosumab dosing intervals of 3 months [40]. Moreover, the duration of pain-free periods between denosumab doses shortened over the course of treatment, while BTM suppression was also sustained. In addition, denosumab treatment has been associated with transient increases in the homogeneity and radiodensity of craniofacial FD lesions on CT and MRI, but these improvements regress after cessation of treatment [44,45]. In a separate case series of 12 patients with FD/MAS treated with denosumab over the course of approximately 15 months, 10 reported a reduction in bone pain along with significant reductions in BTMs [43]. In a case published by our group of a 21-year-old woman with craniofacial FD treated with two doses of denosumab (120 mg) spaced six months apart, there was no reported decrease in pain after either treatment [10], while in a patient with MAS (Patient 1 in this article) whose pain was continuously monitored over 12 weeks, moderate levels of pain persisted during denosumab treatment. At the time of writing this article, two open labeled investigations of denosumab in patients with FD/MAS are active (NCT03571191 and NCT05419050). Taken together, multiple case studies have shown that pain relief in many but not all FD patients can be achieved with denosumab, yet there remains a need for more rigorous testing of denosumab in FD/MAS, particularly in RCTs, while the identification of factors that predict analgesic response or non-response following denosumab treatment is also necessary. Moreover, the adverse effects stemming from the discontinuation of denosumab, for example, hypercalcemia, hypophosphatemia, accelerated loss of bone mineral density, and an increased risk for bone fracture, may limit the use of denosumab [47,48]. Hypocalcemia soon after denosumab treatment induction may also occur [49]. Thus, a risk–benefit assessment and post-denosumab treatment plan are required prior to the start of denosumab therapy.

Calcitonin: Calcitonin is a hormone produced by C-cells in the thyroid, which functions by directly by downregulating the activity of osteoclasts and reducing serum calcium levels [50]. In a case report of a single patient with FD/MAS, intranasal calcitonin was associated with improvements in pain severity at the site of bony lesions and improvements in quality of life [51] (Table 1). In this study, specific type 1 collagen levels were not assessed, but ALP, calcium, and phosphorous levels were unchanged [51]. The ability of calcitonin to reduce bone pain in general is debatable [52]. Further prospective studies of calcitonin are needed to determine its therapeutic utility in FD.

Tocilizumab: Abnormal bone cells in FD/MAS have been shown to secrete interleukin-6 (IL-6), prompting the investigation of tocilizumab, a selective IL-6 inhibitor, as a therapeutic agent in FD/MAS [53]. In a recently completed RCT, 16 patients were randomized to tocilizumab (8 mg/kg IV per month) followed by placebo, or placebo followed by tocilizumab in crossover fashion (Table 1). At the end of the 6-month crossover period, there was no significant change in serum markers of bone turnover or bone pain [54].

**Table 1:** The effect of disease-modifying agents on bone pain, BTM listed by type of study.

Agent	Effect on Pain	Effect on Bone Turnover Markers	Type of Study	Number of Patients (Citation)
Alendronate	No change N/A	Decrease in urine NTX-telopeptide, no change in serum osteocalcin Reduced serum ALP	Phase III RCT Case series	*n* = 40 (Boyce, 2014) [26] *n* = 1 (Wang, 2019) [30]
Pamidronate	Reduced N/A	Reduced urine hydroxyproline, and serum CTX Reduced serum ALP	Phase III trial * Case series	*n* = 20 (Chapurlat, 1997) [27] *n* = 10 (Wang, 2019) [30]
Zoledronate	Reduced N/A	Reduced serum ALP Reduced serum ALP, and CTX	Case report Case series	*n* = 1 (Mansoori, 2010) [29] *n* = 10 (Wang, 2019) [30]
Denosumab	Reduced Reduced Reduced *** Reduced in majority Reduced Reduced Reduced No change	Reduced serum osteocalcin, ALP and CTX Reduced ** Reduced serum P1NP, and urine deoxypyridinoline Reduced serum ALP, P1NP Reduced serum ALP, P1NP, CTX Reduced serum tartrate resistant acid phosphatase Reduced serum CTX -	Case report Case report Case series Case series Case report Case report Case report Case series	*n* = 1 (Eller-Vainicher, 2017 [40] *n* = 1 (Wang, 2014) [41] *n* = 2 (Ganda, 2013) [42] *n* = 12 (Majoor, 2019) [43] *n* = 1 (Raborn, 2020) [44] *n* = 1 (Ikuta, 2021) [45] *n* = 1 (Benhamou, 2014) [46] *n* = 2 (Golden, 2022) [10]
Calcitonin	Reduced	No change in ALP	Case report	*n* = 1 (Fighera, 2017) [51]
Tocilizumab	No change	No change	RCT	*n* = 13 (Chapurlat, 2022) [54]

CTX = C-type 1 collagen telopeptide, NTX = N-telopeptide of type 1 collagen, ALP = alkaline phosphatase, P1NP = propeptide of type 1 procollagen, RCT = randomized control trial. * Open-labeled phase III prospective trial without randomization or a control group. ** Authors did not specify which markers were reduced. *** One patient had a reduction in bone pain.

## 3. Experiences with Pain in Individual FD/MAS Cases

Below, we describe two patients enrolled in our ongoing clinical study of pain in FD/MAS that highlight some of the challenges of treating pain in this disease. This study was approved by the Boston Children’s Hospital (BCH) and the Massachusetts General Brigham, Institutional Review Boards. Patient 1 and Patient 2 provided informed consent prior to their respective involvement in the study. The two cases are highlighted to demonstrate the complexities of treating and alleviating pain on a long-term basis in FD/MAS even though robust therapeutic interventions are employed, such as bisphosphonates, denosumab, and surgical procedures.

Patient 1: A 24-year-old female with MAS. At age 11, she underwent enucleation of aneurysmal bone cyst/giant cell lesion of the mandibular symphysis, followed by adjuvant treatment with three doses of zoledronate. At age 17, she presented with persistent pain in the mandible prompting CT of the head, which showed progressive lytic lesions in the mandibular symphysis associated with soft tissue swelling consistent with a giant cell tumor. Due to side effects (i.e., nausea and vomiting) experienced during the initial bisphosphonate treatment, a second course was not attempted. Instead, denosumab was offered. The patient received her first dose of denosumab (60 mg) one month later, the second dose (30 mg) two months later, and the third dose (30 mg) three months later. Following the third dose, her jaw pain completely resolved but she developed severe nausea, vomiting, and dehydration leading to a hospital admission, where she was found to be profoundly hypercalcemic, and subsequently treated with IV fluids and furosemide.

One year later, she had an interval CT, which showed increased radiolucency in the left sphenoid bone and radiolucent lesion in the left paramedian mandibular symphysis. Over the following two years, she remained without jaw or facial pain but with increasing pain in the right arm and shoulder. Three years later, she presented with persistent pain in the left frontotemporal and right occipital regions associated with severe, debilitating headaches. CT showed an expansile lytic lesion over the left temporal bone with dehiscence. The lesion was aspirated, and fluid studies were negative for infection or cerebrospinal fluid. The pain resolved immediately after sampling, but returned two hours later. After a careful discussion of the risks and benefits, she agreed to a retrial of a three-dose treatment with denosumab.

**Figure 1:**
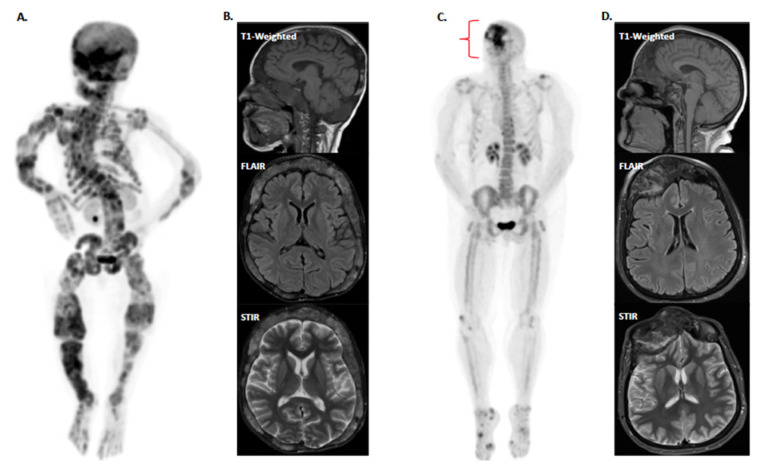
Whole-body ^18^F-NaF PET/CT and non-contrast craniofacial MRI for Patient 1 at age 23 (**A**,**B**) and Patient 2 at age 53 (**C**,**D**). (**A**) There is diffuse radioactive uptake in the axial and appendicular skeleton with pronounced focus in the craniofacial regions indicating polyostotic FD. (**B**) Craniofacial regions with diffuse FD lesions. Skull-based lesions are highlighted. (**C**) There is pronounced ^18^F-NaF uptake in the craniofacial region with limited uptake in the rest of the body. (**D**) Extensive FD affecting the nasal bones and distorting the structure of the sinus cavity. ^18^F-NaF PET = ^18^F-sodium fluoride positron emission tomography, CT = computed tomography, MRI = magnetic resonance imaging, FLAIR = fluid-attenuated inversion recovery, STIR = short tau inversion recovery.

**Figure 2:**
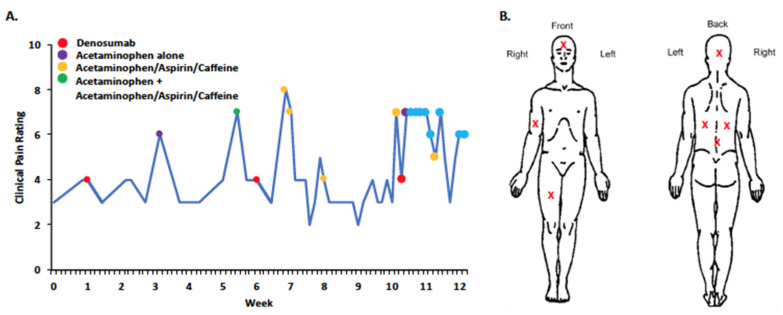
(**A**) The 12-week daily pain diary completed by Patient 1 while undergoing treatment with denosumab at age 23. Average pain was assessed using a 0–10 numerical rating scale and was initiated before the onset of denosumab treatment. (**B**) At baseline and using the BPI, anatomical regions where pain was experienced was identified on a body map. Pain was widespread and present in craniofacial and axial–appendicular locations.

Prior to denosumab treatment, we obtained self-reported baseline pain levels, ^18^F-sodium fluoride positron emission tomography/computed tomography (^18^F-NaF PET/CT) and non-contrast magnetic resonance imaging (MRI) (Figure 1A,B), which showed fibrous lesions affecting multiple structures within the craniofacial and the axial and appendicular skeleton. On the Brief Pain Inventory (BPI) [55], she described pain with a variety of descriptors, including aching, sharp, penetrating, and miserable. Throughout her denosumab treatment she maintained a 12-week daily pain diary (Figure 2). Dose one was given at week one, dose two at week five, and dose three at week 10. Following the first and second doses, her pain scores decreased from 4 to 3 (scale 0 to 10), but increased again to a maximum of 8 one week later. Following the third dose, her reported pain increased from four to seven. During this period, she was also taking acetaminophen and acetaminophen-aspirin-caffeine, which yielded reductions in pain compared to denosumab therapy.

For Patient 1, denosumab provided short-term or periodic pain relief, particularly during her first trial, yet the overall benefit of denosumab was limited due to several treatment-induced adverse effects. While pain did not completely resolve during Patient 1′s retrial of denosumab, on average, her pain did not significantly worsen relative to her baseline levels. Upon post-study follow-up, the patient expressed that she had low expectations for denosumab to decrease her pain at the start of treatment, though she noted that denosumab controlled her FD. Patient 1′s outlook that denosumab will likely not lower her pain may represent a psychological barrier towards achieving pain relief, but this requires further investigation and dialogue with the patient. Her current and main source of pain is localized to her humerus, which showed high levels of ^18^F-NaF uptake prior to denosumab treatment induction, yet the level of uptake was comparable to other FD lesions such as those within craniofacial locations.

Patient 2: A 53-year-old woman with craniofacial FD/MAS involving the bilateral frontal bones, sphenoid, ethmoid, and clivus. She was diagnosed with FD in her teens during a workup of recurrent sinus infections. At age 21, during her first pregnancy, she developed visual disturbances leading to loss of vision in her right eye within a week. She underwent emergent surgical removal of FD tissue around her optic nerve, which restored her eyesight but resulted in frequent headaches. Two years later, she underwent a second surgical removal of FD tissue because of worsening headaches, but developed severe migraines afterward. Alendronate did not relieve her pain, but a combination of analgesics, administered through a pain specialist, achieved reasonable pain control (medication details not known). Her second pregnancy was uneventful. However, she developed right-sided facial pain and frequent sinus infections over the years.

**Figure 3:**
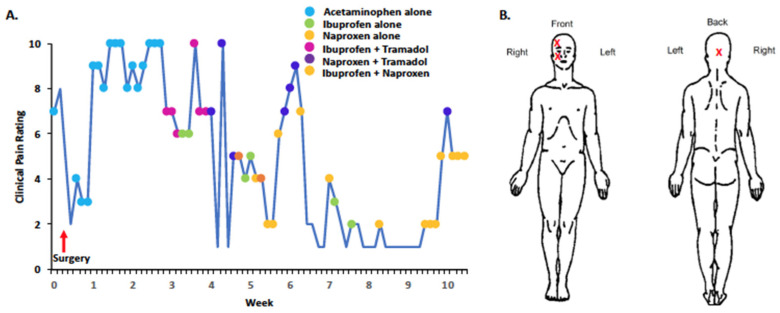
(**A**) The 10-week daily pain diary completed by Patient 2 at age 53. Average pain was assessed using a 0–10 numerical rating scale and was initiated before surgical treatment to lessen craniofacial FD lesions. (**B**) At baseline and using the BPI, anatomical regions where pain was experienced were confined to craniofacial areas.

A craniofacial CT at age 47 showed extensive lytic lesions affecting bilateral facial bones. This prompted a third surgery involving the removal of FD tissue from the sinus cavity. Pain relief lasted for less than a year and headaches and sinus infections recurred. Repeat CT showed expensive sclerotic/cystic bony changes involving frontal, ethmoidal, and sphenoid sinuses with distortion of the sinus anatomy, which led to a fourth surgery to remove FD tissue between the sphenoid ostia, right orbital, ethmoid, and frontal regions. Unfortunately, this did not provide symptom relief, and she continued to experience ongoing, pulsating headaches moving from the right to the top of the head. Given her persistent symptoms, she was offered zoledronate and/or denosumab therapy, but neither medication had been initiated at the time of writing this manuscript. She enrolled in our study before her latest surgery. On the BPI, she described her pain as aching, stabbing, penetrating, miserable, and unbearable. Initial ^18^F-NaF PET/CT and MRI scans (Figure 1C,D) showed extensive craniofacial FD with postsurgical changes from prior removal of FD tissue in the nasal cavities. We began to monitor her pain scores prior to the fourth surgery at week 0 (Figure 3). The pain scores fell from seven to three one week after surgery while taking acetaminophen, but quickly rebounded. The patient was also prescribed oxycodone at the time of her surgery. Throughout the 10-week period her pain scores gradually decreased with acetaminophen, naproxen, ibuprofen, and tramadol (Figure 3).

At post-study follow-up, Patient 2 communicated that her pain once again became debilitating. Despite experiencing severe bouts of pain, the patient noted a strong hesitancy for using any opioid-based therapies, and only used tramadol sparingly given the risk of developing addiction. Patient 2 continued to express concerns regarding potential side effects associated with bisphosphonates and denosumab. Four months after the study period ended, she attempted acupuncture, which reduced her pain scores to 1/10. Her pain remained at a very low level during her acupuncture treatment. The implementation of alternative or non-pharmacological pain treatment approaches such as acupuncture may complement pharmacological (e.g., bisphosphonates or denosumab) or surgical treatment approaches in FD/MAS.

## 4. Ameliorating Pain in FD/MAS: Key Needs and Future Directions

The currently available pharmacological treatments for FD/MAS remain limited to a handful of agents—mainly bisphosphonates and denosumab, with additional analgesics. In the case of bisphosphonates and denosumab, the justification for their use in FD/MAS for mitigating skeletal lesions or reducing pain is based primarily on single-site experiences of these therapies, their open-label use and retrospective treatment evaluation. Unfortunately, rigorous RCTs are lacking, with the exception of one trial for alendronate and one for tocilizumab in FD/MAS [26,54], which are especially critical for the assessment of any potential analgesic treatment. To evaluate the analgesic properties of any therapy, placebo-controlled trials are necessary considering the strong behavioral as well as biological responses placebo alone can induce (e.g., activation of the brain’s endogenous opioid system) [56,57,58]. Moreover, while bisphosphonates and denosumab are disease-modifying, their clinical benefit can be temporary and associated, in some cases, with severe side effects. An additional limitation to the use of bisphosphonates in reproductive-age women is the long duration for which the medication remains in the bone, and it could potentially be teratogenic to offspring in the event of pregnancy even years after bisphosphonate treatment [59]. Denosumab may be beneficial in cases of FD in women of reproductive age [10], as it is a monoclonal antibody to RANK-L that only persists in the body for 6 months after each dose. Additional side effects of denosumab include rebound hypercalcemia following denosumab treatment cessation, nausea, and vomiting, which can require hospitalization, while bisphosphonates are associated with hypocalcemia and esophagitis, among other adverse effects. Corrective surgery, while frequently employed in FD/MAS, does not consistently provide sustained pain relief. The shortcomings of surgery and existing pharmaceutical options that modify bone turnover pathways suggest the need for novel therapeutic targets.

## 5. Novel Targets for Treating Bone Pain in FD/MAS: A Potential Role for ASICs and GDNF

**Figure 4:**
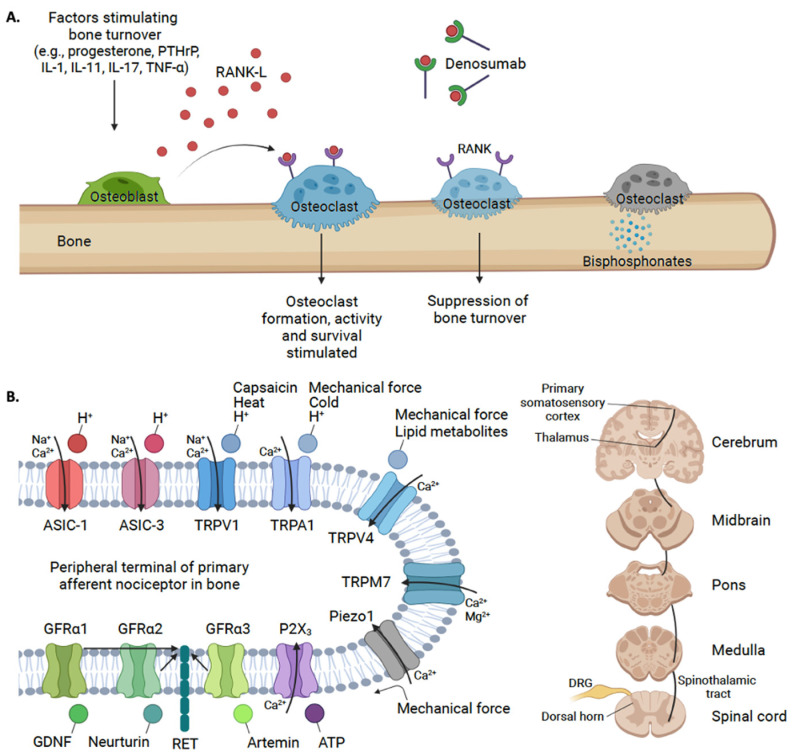
(**A**) RANK-L binds to RANK on osteoclasts resulting in differentiation and maturation of osteoclasts and subsequent bone resorption [97]. Denosumab binds to RANK-L and inhibits its action, and prevents osteoclast recruitment and maturation, resulting in slowed bone resorption [35]. Bisphosphonates prevent bone resorption by binding to hydroxyapatite and inhibiting osteoclast function [98]. (**B**) Nociceptive signaling molecules on primary sensory neurons innervating skeletal tissue can include the transient receptor potential (TRP) family channels, acid-sensing ion channels (ASICs), mechanosensitive ion channels (Piezo1), purinergic receptors (P2X_3_), and Glial cell line-derived neurotrophic factor (GDNF)/ GDNF Family Receptor Alpha (GFRα) receptors [72]. Bone pain may involve the activation and sensitization of neurons via Glial cell line-derived neurotrophic factor (GDNF)/GFRα1, neurturin/GFRα2, and artemin/GFRα3 signaling pathways [75,95]. The percephin/GFRα4 complex is not included. When peripheral afferent neurons in bone tissue, with their cell bodies located in the dorsal root ganglion (DRG), are activated, nociceptive signals are carried via the spinothalamic and ascending pain pathways to convey pain signals to various regions in the brain [99,100]. Note that the activation of GFRα receptors may yield a potential increase in synaptic channel or receptor expression and/or sensitization (Created with BioRender.com, accessed on 5 August 2022).

The development of FD lesions is driven by defective processes involving osteoclasts and osteoblasts—pathological processes that are hypothesized to also facilitate the generation of pain in FD/MAS or bone pain in general [60,61,62,63] (Figure 4). Nonetheless, it remains unclear which neurobiological mechanism(s) may underpin FD/MAS pain. In prior work, bone remodeling and inflammatory processes have been suggested to generate bone pain in FD/MAS [64]. Bone tissue is heavily innervated by myelinated and unmyelinated sensory nerves, which express toll-like receptors (TLR), transient receptor potential (TRP) ion channels, receptor tyrosine kinases (RET) and neurotrophic receptor tyrosine kinase type 1 (TrkA), among other receptors and channels [65,66]. Innervation is especially dense within the periosteum and bone marrow compartments, with enhanced innervation occurring proximal to active bone remodeling sites [67,68,69]. With significant bone turnover, mechanosensitive ion channels, such as Piezo1, may be activated when sensory nerve fibers are distended from mechanical pressure [70,71,72]. Some bone pain is postulated to be driven by osteoclast-induced acidosis, which may activate adenosine triphosphate (ATP) gated ion channels; purine receptor (P2X_3_), transient receptor potential vanilloid (TRPV) channels with TRPV1, TRPA1 and TRPV4. The transient receptor potential cation channel, subfamily M and member 7 (TRPM7) channels have been reported to be present in bone, but only TRPV1 and transient receptor potential ankyrin 1 (TRPA1) respond to pH changes [73,74]. Decreasing pH will activate acid-sensing ion channels (ASICs), including ASIC-1 and ASIC-3 [61,70] which may also contribute to bone pain (Figure 4). Moreover, there is accumulating evidence for a role of the Glial cell-derived neurotrophic growth factors (GDNF) family of ligands and their associated receptors in nociceptive signaling through bone afferent neurons [75]. Below, we emphasize the roles of ASICs and GDNFs, which are hypothesized to play a role in driving pain in FD/MAS and may serve as novel analgesic targets for this and other rare bone diseases.

ASICs: Osteoclast-induced bone resorption is dependent on acidification of the extracellular matrix, in particular the interface between the osteoclast cell membrane and the bone surface [76,77]. Extracellular pH is decreased by the efflux of H^+^ (protons) through ion pumps on the surface of osteoclasts [78]. The acidic pH in the extracellular space both stabilizes alkaline bone mineral substrate and activates lysosomal enzymes, which degrade bone [79]. While extracellular acidification is physiologic and necessary for normal osteoclast function, excess acidification has been implicated in chondrocyte apoptosis [80,81] and rheumatoid arthritis [82]. We argue that the resultant bone pain seen in FD/MAS is driven, at least in part, by the excessive acidification of the extracellular matrix leading, to the activation of ASICs on the surface of nociceptors. ASIC-1a in particular is highly expressed in neurons and is an important mediator of pain [83,84]. Extracellular acidosis during bone remodeling can activate ASICs on the surface of nociceptive fibers innervating musculoskeletal tissue or compartments [84,85,86]. In preclinical models of bone cancer, ASIC-1 expression alone was increased in the dorsal root ganglion (DRG), which may play a role in inducing hyperalgesia [87]. This direct activation of nociceptors by osteoclasts via ASICs is implicated in the development of arthritic bone pain [82,88,89]. Separately, the blockade of ASIC-3 signaling reversed the mechanical hypersensitivity in mice that previously demonstrated allodynia [89]. In addition, transgenic mice with rheumatoid arthritis that were deficient in ASIC-3 did not develop mechanical hypersensitivity [89]. Thus, bone pain in FD may be influenced by increased ASIC-1 and 3 signaling (Figure 4).

GDNFs: Subsets of the GDNF pathway have been implicated in the development of bone pain [75]. The GDNF family of ligands includes GDNF (binds GFRα1), neurturin (binds GFRα2), artemin (binds GFRα3), and persephin (binds GFRα4) [90]. GFRα1, GFRα2, and GFRα3 are expressed on nociceptive sensory as well as sympathetic neurons and ganglia [91,92], while GFRα4 is not found in peripheral sensory neurons [93]. GFRα3, in particular, shares nociceptive cell markers, such as calcitonin gene-related peptide (CGRP), TRPV1, TrkA, and RET [94]. Bone pain may involve the activation and sensitization of neurons via GDNF/GFRα1, neurturin/GFRα2, and artemin/GFRα3 signaling pathways [75,95]. In animal studies, GDNF, neurturin, and artemin have been implicated in inflammatory bone pain via activation of the nerve growth factor (NGF (ligand for TrKA))-sensitive neurons [75]. In an animal model, the overexpression of artemin led to, on one hand, the increased density of neurons in the dorsal root ganglia and the expression of GFRα3, TrkA, TRPV1, and TRPA1, while on the other, a decreased expression of ASICs [96]. We suggest that the interrogation of GDNF pathways, particularly artemin-GFRα3 signaling, in preclinical models of FD may provide insights into the role of the associated receptors and ligands, as well as inflammatory pain mechanisms, in FD/MAS [18].

## 6. Conclusions

From a pain treatment perspective, current therapies for FD/MAS have shown analgesic benefits alongside adverse effects in case series and retrospective studies. RCTs are necessary for FD/MAS, especially in light of the prominent placebo effect in clinical pain trials. Although treatments such as bisphosphonates, denosumab, and surgery can provide pain relief to patients with FD/MAS, we propose a more rigorous evaluation of these approaches, and a firmer biological and behavioral understanding of pain mechanisms active in FD/MAS should be pursued in parallel.

## 7. Design of Review

A review of primary studies (e.g., case reports, or clinical trials) that have reported on pharmacologic treatments for FD/MAS was performed (see Table 1). We included disease-modifying agents that act directly or indirectly in some way on osteoclast function ((i) bisphosphonates, (ii) denosumab, (iii) calcitonin and (iv) tocilizumab) and excluded over-the-counter analgesics (e.g., non-steroidal anti-inflammatory drugs, acetaminophen) and electrolyte supplements such as vitamin D and calcium. We conducted a comprehensive search of published literature, clinical trials, cohort studies, case reports, review articles and systematic reviews for disease-modifying agents in fibrous dysplasia/McCune–Albright Syndrome. The studies selected for inclusion in this review were accessed through PUBMED, Google Scholar, CENTRAL, and MEDLINE from 1970 to 2022. The search terms included subject headings, text words and keywords with the following terms: “fibrous dysplasia/McCune–Albright Syndrome”, “bone pain”, “analgesia”, “disease modifying”, “bisphosphonates”, “denosumab”, “calcitonin”, “tocilizumab”, “mithramycin”, “Glial-derived neurotrophic factor”, “GDNF”, “GFRα1-4”, “neurturin”, “artemin/GFRα3”, and “persephin”.

## Data Availability

Anonymized data not published within this article will be made available by request from any qualified investigator.
